# Supplementary material for: Empirical methods for controlling false positives and estimating confidence in ChIP-Seq peaks
Source: BMC Bioinformatics. 2008 Dec 5;9:523. doi: 10.1186/1471-2105-9-523 (PMC2628906; doi:10.1186/1471-2105-9-523)
Supplement: Additional File 1 — A variety of html documents from the USeq web site detailing the available applications, their best usage, output file type descriptions, command line menus, etc. [file 1471-2105-9-523-S1.zip › USeqUserGuides/JavaDoc.zip!/JavaDoc/util/gen/LinearRegression.html]

LinearRegression


|  |  |  |
| --- | --- | --- |
| **Overview** \| **Package** \| **Class** \| **Use** \| **Tree** \| **Deprecated** \| **Index** \| **Help** | |  |
| **PREV CLASS**   **NEXT CLASS** | **FRAMES**    **NO FRAMES**     **All Classes** |  |
| SUMMARY: NESTED \| FIELD \| CONSTR \| METHOD | DETAIL: FIELD \| CONSTR \| METHOD |  |


---


## util.gen Class LinearRegression

```
java.lang.Object
  util.gen.LinearRegression
```

---

``` public class LinearRegression extends java.lang.Object ```

For calculating a simple linear regression.
Adapted from http://www-stat.stanford.edu/~naras/java/course/lec2/
Balasubramanian Narasimhan, Stanford

---

**Constructor Summary**

`LinearRegression(double[] x,
double[] y)`


| **Method Summary** | |
| --- | --- |
| `double` | `calculateX(double y)`             Calculate X given Y. |
| `double` | `calculateY(double x)`             Calculate Y given X. |
| `double` | `getIntercept()` |
| `java.lang.String` | `getModel()`             Returns Y=mX+b with full precision, no rounding of numbers. |
| `java.lang.String` | `getRoundedModel()`             Returns Y=mX+b |
| `double` | `getRSquared()` |
| `double` | `getSlope()` |
| `double[]` | `getX()` |
| `static double` | `interpolateX(double x1, double y1, double x2, double y2, double fixedY)`             Return approximated X value, good for a single interpolation, multiple calls are inefficient! |
| `static double` | `interpolateY(double x1, double y1, double x2, double y2, double fixedX)`             Return approximated Y value, good for a single interpolation, multiple calls are inefficient! |
| `static void` | `main(java.lang.String[] args)`             An example. |
| `void` | `nullArrays()`             Nulls the x and y arrays. |

**Methods inherited from class java.lang.Object**

`equals, getClass, hashCode, notify, notifyAll, toString, wait, wait, wait`

**Constructor Detail**

### LinearRegression

```
public LinearRegression(double[] x,
                        double[] y)
```


**Method Detail**

### main

```
public static void main(java.lang.String[] args)
```

:   An example.

---


### interpolateY

```
public static double interpolateY(double x1,
                                  double y1,
                                  double x2,
                                  double y2,
                                  double fixedX)
```

:   Return approximated Y value, good for a single interpolation, multiple calls are inefficient!

---


### interpolateX

```
public static double interpolateX(double x1,
                                  double y1,
                                  double x2,
                                  double y2,
                                  double fixedY)
```

:   Return approximated X value, good for a single interpolation, multiple calls are inefficient!

---


### getSlope

```
public double getSlope()
```

---


### getIntercept

```
public double getIntercept()
```

---


### getRSquared

```
public double getRSquared()
```

---


### getX

```
public double[] getX()
```

---


### getModel

```
public java.lang.String getModel()
```

:   Returns Y=mX+b with full precision, no rounding of numbers.

---


### getRoundedModel

```
public java.lang.String getRoundedModel()
```

:   Returns Y=mX+b

---


### calculateY

```
public double calculateY(double x)
```

:   Calculate Y given X.

---


### calculateX

```
public double calculateX(double y)
```

:   Calculate X given Y.

---


### nullArrays

```
public void nullArrays()
```

:   Nulls the x and y arrays. Good to call before saving.


---


|  |  |  |
| --- | --- | --- |
| **Overview** \| **Package** \| **Class** \| **Use** \| **Tree** \| **Deprecated** \| **Index** \| **Help** | |  |
| **PREV CLASS**   **NEXT CLASS** | **FRAMES**    **NO FRAMES**     **All Classes** |  |
| SUMMARY: NESTED \| FIELD \| CONSTR \| METHOD | DETAIL: FIELD \| CONSTR \| METHOD |  |


---
